# Dermal microfilariae of dogs, jackals and cats in different regions of Iran

**DOI:** 10.1186/s13071-021-05141-2

**Published:** 2022-01-20

**Authors:** Alireza Sazmand, Zahra Bahiraei, Farzad Nemati, Giada Annoscia, Marcos Antonio Bezerra-Santos, Hassan Nayebzadeh, Amir Masoud Salemi, Seyed Mahmoud Mousavi, Seyed Mahmoud Sadjjadi, Domenico Otranto

**Affiliations:** 1 Department of Pathobiology, Faculty of Veterinary Science, Bu-Ali Sina University, 6517658978 Hamedan, Iran; 2 Zoonotic Diseases Research Center, School of Public Health, Shahid Sadoughi University of Medical Sciences, 8915173160 Yazd, Iran; 3 Department of Veterinary Medicine, University of Bari Aldo Moro, Str. prov. per Casamassima km 3, Valenzano, 70010 Bari, Italy; 4 Department of Pathobiology, Faculty of Veterinary Medicine, Lorestan University, 6815144316 Khorramabad, Iran; 5 Department of Parasitology and Mycology, School of Medicine, Shiraz University of Medical Sciences, Shiraz, Iran; 6 Research Center of Basic Sciences in Infectious Diseases, Shiraz University of Medical Sciences, Shiraz, Iran

**Keywords:** *Cercopithifilaria bainae*, Dermal microfilariae, *Onchocerca lupi*, Dog, Neglected, Vector-borne

## Abstract

**Background:**

Due to the complexity of retrieving skin-dwelling microfilariae, filarioids of dogs presenting dermal microfilariae (e.g. *Cercopithifilaria* spp., *Onchocerca lupi*) are relatively unknown compared to *Dirofilaria* spp*.* and *Acanthocheilonema* spp*.* whose microfilariae circulate in the blood. Although *Cercopithifilaria* spp. and *O. lupi* filarioids are distributed worldwide, there is a paucity of information on their occurrence in Iran. The aim of this study was to investigate these filarioids in a large population of dogs from different regions of Iran.

**Methods:**

From October 2018 to September 2020, skin biopsies were obtained from dogs housed in shelters (*n* = 557) and privately owned dogs (*n* = 26) in seven provinces of Iran (Hamedan, Kermanshah, Yazd, Mazandaran, Khuzestan, Lorestan, Esfahan), as well as from three road-killed jackals (*Canis aureus*) and three cats (*Felis catus*) in Hamedan province. The skin biopsies were first soaked in saline solution at room temperature overnight, and examined for dermal microfilariae under the microscope. Positive skin specimens and sediments were tested by PCR for a 304-bp region of the mitochondrial cytochrome *c* oxidase subunit 1 (*cox*1) gene and amplicons were sequenced.

**Results:**

Microfilariae of *Cercopithifilaria* spp. were found in skin biopsies of 32 of the 583 (5.5%) dogs tested, with infection rates of up to 25% in Kermanshah. No microfilariae were recovered from skin biopsy samples collected from dogs in Khorramabad and Ahvaz, nor from the examined jackals and cats. None of the privately owned dogs were found to be infected. Morphologic and morphometric characteristics of the microfilariae were consistent with *C. bainae*. Eighteen skin samples were positive for the *cox*1 gene, of which 15 sequences showed a nucleotide identity of 100% and three of 93.4% with the reference sequence of *C. bainae* available in GenBank (haplotype I; GenBank accession number: JF461457).

**Conclusions:**

The data from this study broadens current knowledge on the geographical distribution of *C. bainae* in dogs in Middle Eastern countries. Further studies on different wild canine species in the country (e.g. jackal, fox, wolf) could provide further information on the epidemiology of these filarioids. A particular focus should be put on zoonotic *O. lupi* given the reports of its presence in human patients from this country.

**Graphical Abstract:**

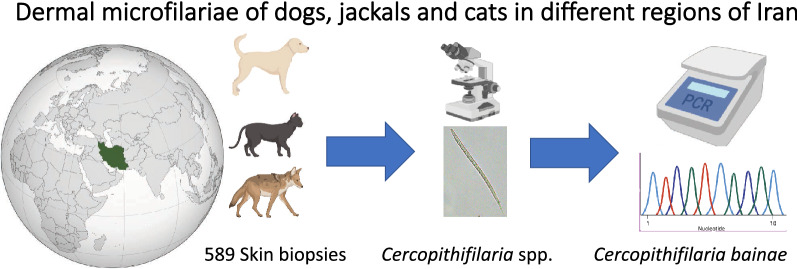

**Supplementary Information:**

The online version contains supplementary material available at 10.1186/s13071-021-05141-2.

## Background

Until 15 years ago, filarial nematodes releasing microfilariae into the haematic circulation, such as *Dirofilaria immitis*, *Dirofilaria repens*, *Acanthocheilonema reconditum*, *Acanthocheilonema dracunculoides* and *Brugia malayi*, were considered to be the most prevalent filarioids affecting dogs [1]. Conversely, those which present dermal microfilariae, such as *Cercopithifilaria* spp. and *Onchocerca lupi*, have been largely neglected by parasitologists and veterinary practitioners mainly due to the difficulties in retrieving microfilariae in the skin and to the limited number of clinical conditions they cause [2, 3]. Members of the genus *Cercopithifilaria* are parasites of cercopithecus monkeys, ruminants, rodents, lagomorphs, marsupials, monotremes and carnivores [4, 5]. In the past decade, three of the 29 species of *Cercopithifilaria* (i.e. *Cercopithifilaria bainae*, *Cercopithifilaria grassii* and *Cercopithifilaria* sp. II sensu Otranto et al. 2013) have been reported in domestic dogs (*Canis lupus familiaris*) and red foxes (*Vulpes vulpes*) of Europe and the Americas [6, 7]. Infections with *Cercopithifilaria* spp. are generally considered to be minimally pathogenic as most of the infected dogs do not present physical abnormalities that could be associated to the presence of microfilariae in the skin. However, dermatitis [7, 8], diffused chronic polyarthritis without any other apparent cause [9] and gross skin lesions [10] have been reported in infected dogs. Recently, an infected dog with a giant cutaneous cyst in the lumbosacral region with numerous microfilariae of *C. bainae* was also reported [11], suggesting a further pathogenic role of this species in infected dogs. *Rhipicephalus sanguineus* sensu lato (s.l.) is considered to be the competent biological vector of these nematodes [12, 13] although DNA of *Cercopithifilaria* spp. has been also detected in *Rhipicephalus turanicus* and different genogroups within *R. sanguineus* s.l. from different countries in Europe, Africa, Asia and Australia [14].

Microfilariae of the zoonotic nematode *Onchocerca lupi*, which infects dogs, wolves, cats, coyotes and humans, can also be detected in skin biopsies of the infected carnivores [15]. Infected dogs may display acute or chronic ocular diseases [16] or be completely asymptomatic due to the localisation of adult nematodes in the retrobulbar space of the eye [17]. The parasite’s life-cycle, the incubation time and the prepatent period are not fully understood, but black flies of the genus *Simulium* are suspected to be involved as intermediate hosts [15]. Since the first report of zoonotic infection by *O. lupi* in humans [18], infection has been diagnosed in more than 20 patients in Iran, Turkey, Tunisia, Greece, Hungary, Germany and the USA [19]. Human patients manifest an invasive disease featured by spinal, orbital, ocular and subdermal nodules [20–22].

Canine and feline infection with dermal microfilariae has been reported from different European and American countries [23]. However, although human infection with *O. lupi* has been confirmed in Iran and neighbouring Turkey [18, 22, 24] no information is available on *O. lupi* infection in dogs.

Dogs affected by *Cercopithifilaria* spp. and *O. lupi* sometimes do not display any clinical signs [25, 26]. In the context of this background, together with the presence of *R. sanguineus* s.l. (i.e. vectors of *Cercopithifilaria* spp.) in Iran [27–31], we decided to investigate dermal microfilariosis in dogs from different regions of the country, aiming to improve scientific knowledge on the distribution of these little known filarioids.

## Methods

### Study area

**Fig. 1:**
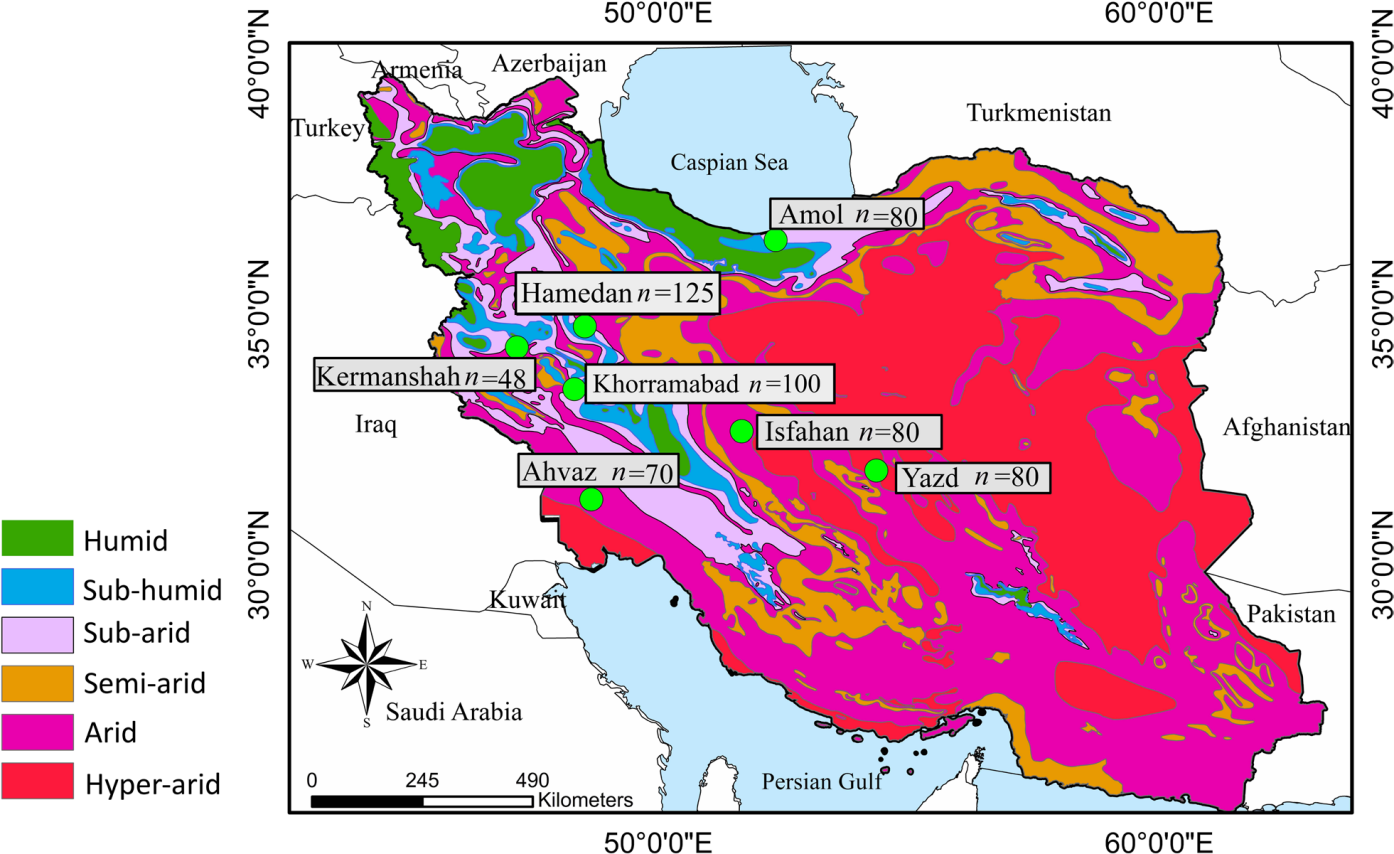
Map of Iran showing the sampling localities and the number of dogs examined in each site

**Fig. 2:**
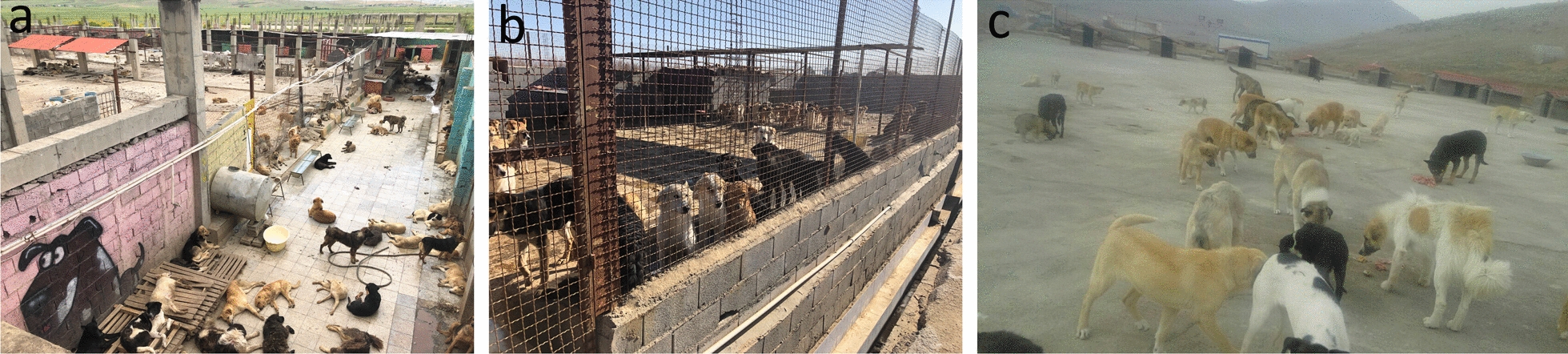
Shelters in Kermanshah (**a**), Yazd (**b**) and Lorestan (**c**) provinces where dogs were sampled

The study was carried out in seven provinces in Iran (i.e. Hamedan, Kermanshah, Yazd, Mazandaran, Khuzestan, Lorestan and Esfahan (Fig. 1). Most of the samples (537/538; 92.1%) were taken from animal shelters that had been built by non-governmental organisation (NGOs) to feed stray dogs. Dogs in these shelters had been rescued from all of the seven provinces (Fig. 2).

### Collection of specimens and microscopic examination

From October 2018 to September 2020, 583 dogs aged > 6 months (537 dogs from the chosen shelters and 46 privately owned dogs) were sampled at seven sampling sites (Fig. 1). Clinical examination was performed, and anamnestic data were recorded for each dog sampled; the dogs sampled had not been treated with endoparasiticides for at least 1 month prior to the sampling. During the study period, three road-killed jackals (*Canis aureus*) and three cats (*Felis catus*) in Hamedan were also biopsied.

Skin biopsies (about 0.5 × 0.5 × 0.5 cm) were taken using disposable scalpels from the inter-scapular region and soaked in 1 ml saline solution (NaCl 0.9%) at room temperature for 10 h or overnight. A drop of sediment from the soaked skin sample was then placed on a glass slide and observed under the light microscope (2 fields of 18 × 18-mm coverslip each) at a magnification of ×100. Dermal microfilariae were counted and identified based on morphological features described previously [10, 25]. Photomicrographs were taken of microfilariae using an Olympus CX41 microscope (Olympus Corp., Tokyo, Japan) equipped with a DP25 camera, and the microfilariae measured. For permanent mounting of microfilariae, the coverslips of positive slides were removed, and then both slides and coverslips were fixed with methanol, then air-dried and stained with Giemsa stain as described previously [32]. Upon diagnosis of microfilariae by microscopy, the skin tissue was transferred into clean microtubes and stored at − 20 °C for molecular analysis.

### Molecular procedures

Genomic DNA was extracted from all skin samples and sediments using a commercial kit (DNeasy^®^ Blood & Tissue kit; Qiagen, Hilden, Germany), according to the manufacturer’s instructions. A 304-bp region of the mitochondrial cytochrome *c* oxidase subunit 1 (*cox*1) gene was amplified using the CbCox1F/NTR primer pair [33]. Amplicons were visualised by electrophoresis in a 2% agarose gel stained with GelRed (VWR International PBI, Milan, Italy) on a GelLogic 100 gel documentation system (Kodak, Rochester, NY, USA). Amplicons were then purified and sequenced in both directions using the same primers as those for the PCRs, by the Big Dye Terminator v.3.1 Cycle Sequencing Kit in a 3130 Genetic Analyzer (Applied Biosystems, Thermo Fisher Scientific, Foster City, CA, USA). Sequences were edited and compared to those available in the GenBank™ dataset by Basic Local Alignment Search Tool (BLAST) analysis (https://blast.ncbi.nlm.nih.gov/Blast.cgi). Phylogenetic relationships were inferred using the Maximum Likelihood (ML) method based on the Tamura–Nei model [34], with the Gamma distribution (+G) used to model evolutionary rate differences among sites selected by the best-fit model [35]. Evolutionary analysis was conducted on 1000 bootstrap replications using MEGA5 software [36]. A homologous sequence from *Litosomoides yutajensis* (GenBank accession number: AJ544869) was used as the outgroup.

### Statistical analysis

Exact binomial 95% confidence intervals (CIs) were established for proportions. The chi-square (*χ*^2^ ) test was used to compare proportions, with a probability value < 0.05 regarded as statistically significant. Analyses were performed using Microsoft Office Excel ver. 16.37 (Microsoft Corp., Redmond, WA, USA).

## Results

Of the 583 dogs from whom skin biopies were taken and examined, 32 (5.5%; 95% CI 5.47–5.51) from five regions (*n* = 12 Kermanshah; *n* = 7 from Hamedan; *n* = 7 from Yazd; *n* = 3 from Amol; *n* = 3 from Esfahan) were positive for microfilariae. Biopsies taken from dogs sampled in Ahvaz and Khorramabad (*n* = 170) all tested negative for microfilariae based on microscopic examination. None of the privately owned dogs nor the jackals and cats were found to be infected. No statistical association was found between *Cercopithifilaria* spp. infection in dogs and sex (*χ*^2^ = 1.43, *df* = 1, *P* = 0.23) or age (*χ*^2^ = 0.49, *df* = 3, *P* = 0.92) (Table 1). At clinical examination, 69 dogs (11.8%) showed ocular clinical signs, such as mucosal discharges, conjunctivitis and keratitis or ulcers.

**Table 1:** Number and percentage of dogs positive for *Cercopithifilaria* spp. (*n* = 583) according to sex, age and sampling area

Variables	*Cercopithifilaria* infection^a^
Sex	
Male	16/207 (7.7%; 7.69–7.77)
Female	14/368 (3.8%; 3.78–3.82)
Unknown	2/8 (25%)
Age (years)	
< 1	2/49 (4.1%; 4.02–4.14)
1–3	19/328 (5.8%; 5.77–5.82)
3–5	5/105 (4.8%; 4.72–4.80)
> 5	5/96 (5.2%; 5.16–5.25)
Unknown	1/5 (20%)
Geographical origin	
Kermanshah	12/48% (25; 24.87–25.13)
Yazd	7/80 (8.7%; 8.69–8.81)
Hamedan	7/125 (5.6%; 5.2–6.0)
Amol	3/80 (3.7%; 3.71–3.79)
Esfahan	3/80 (3.7%; 3.71–3.79)
Ahvaz	0/70
Khorramabad	0/100
Total	32/583 (5.5%; 5.47–5.51)

^a^Infection values are presented as the number of positive skin samples/total number of skin samples tested, with the percentage and 95% confidence interval given in parentheses

**Fig. 3:**
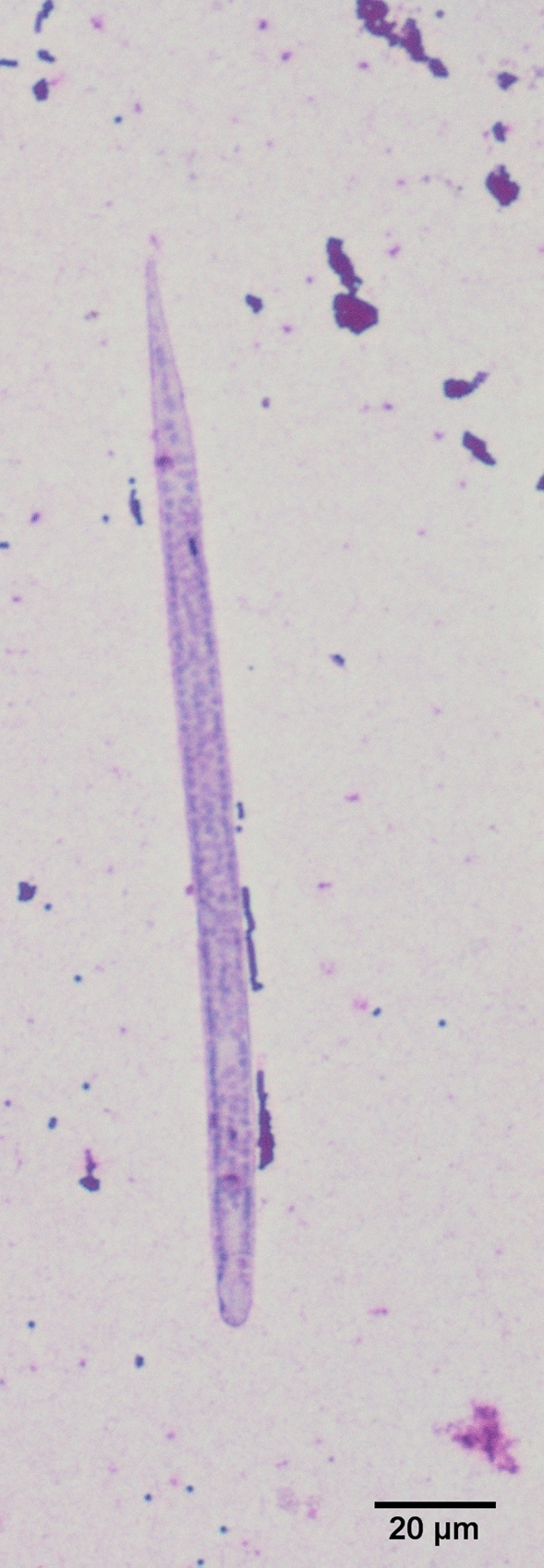
Microfilaria of *Cercopithifilaria bainae* stained with Giemsa. Note the rounded head, short body and tip-pointed tail of the microfilaria (scale bar: 20 μm)

**Fig. 4:**
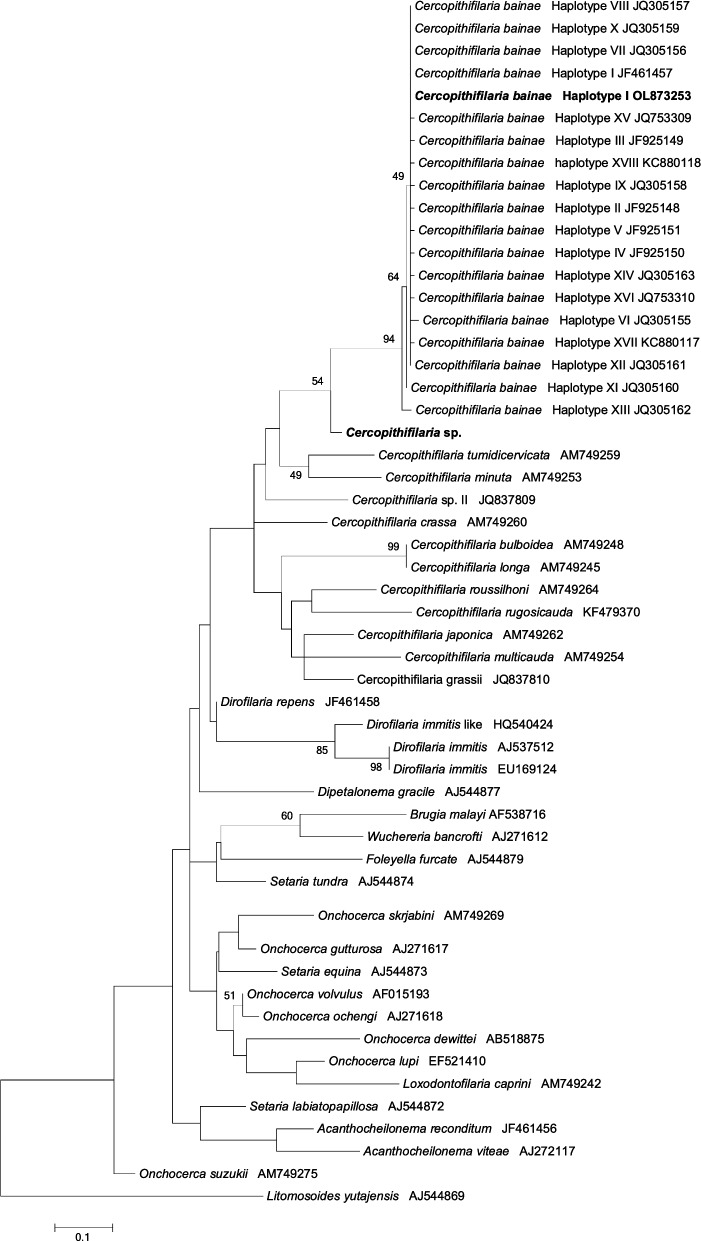
Phylogenetic relationship of *Cercopithifilaria* spp. sequences detected in this study (in bold) and other filarioid nematodes available from GenBank based on a partial sequence of the *cox*1 gene. Evolutionary analysis was conducted on 1000 bootstrap replications using a maximum likelihood method and Tamura–Nei model to model evolutionary rate differences among sites selected by best-fit model. A homologous sequence from *Litosomoides yutajensis* (GenBank accession number: AJ544869) was used as the outgroup

All of the microfilariae were identified as *C. bainae*, with mean length and width of 172.32 and 6.77 µm, respectively, a rounded head and a short dorsoventrally flattened body (Fig. 3, Additional file 1: Video S1). A maximum number of five microfilariae were counted in one sediment. Molecular testing showed that 18 skin samples (56.2%) were positive for the *cox*1 gene. Of these positive samples, 15 sequences showed a nucleotide identity of 100% with the reference sequence of *C. bainae* haplotype I (GenBank accession number: JF461457) and the remaining three showed 93.4% similarity (Fig. 4). The nucleotide sequences of *Cercopithifilaria* sp. identified in this study were deposited in GenBank (accession number: OL873253).

## Discussion

Our survey of *C. bainae* infection in dogs from different regions of Iran indicates that this filarioid is more widespread than previously thought. However, the presence of *Cercopithifilaria* spp. in dogs from Iran could be also inferred by the fact that *R. sanguineus* s.l. is distributed in that region [27]. More specifically, up to 5.5% of the dogs tested were infected with *C. bainae*. In previous studies, infection rates of 9.4% and 13.9% were reported in Italy [1, 37], 21.6% in Spain [1], 4.3% and 13.6% in Greece [1, 26], 9.8% in Portugal [38], 2.6% in Romania [39], 1% in Brazil [40] and 2.6% in USA [41]. A similar variation was found in our survey, with the infection rates in dogs varying according to the study regions, with the highest prevalence of infection (25%) in Kermanshah and no cases in Ahvaz and Khorramabad. This pattern is similar to that observed in dogs from Italy, where the prevalence of *C. bainae* ranged from 7.7% in Sardinia to 13.3% in Sicily [1, 37]. The prevalence of this filarioid has also been reported to vary in Greece, between 2% and 30.8% depending on the sampling location [26]. Of note, infection in the sampled dogs in the different regions could not be associated with climate, elevation above sea level or average annual precipitation (Table 2). It has been hypothesised that *Cercopithifilaria* spp. might be more common in warmer areas [39].

**Table 2:** *Cercopithifilaria* infection rate in dogs included in the study according to geographical location and climatic parameters

Study area	*Cercopithifilaria* infection rate (%)^a^	Coordinates	Elevation above sea level (m)	Climate	Average annual precipitation (mm)	Average annual temperature (°C)
Kermanshah	25	34.3277°N, 47.0778°E	1358	Warm and temperate	437	13.3
Yazd	8.7	31.8974°N, 54.3569°E	1230	Hot and arid	55	18.9
Hamedan	5.6	34.7989°N, 48.5150°E	1823	Cold semi-arid	384	11.3
Amol	3.7	36.4676°N, 52.3507°E	96	Mild and humid	829	15.9
Esfahan	3.7	32.6539°N, 51.6660°E	1590	Desert	97	16.7
Khorramabad	0	33.4647°N, 48.3390°E	1147	Mediterranean	511	17.2
Ahvaz	0	31.3183°N, 48.6706°E	17	Hot and humid	210	24.9

^a^(Number of biopsies positive for *Cercopithifilaria bainae*/total number of biopsies) × 100

In this study, *C. bainae* was the only filarioid species detected. To date, co-infection of *C. bainae* with other *Cercopithifilaria* spp. has been reported in animal populations of Italy with *Cercopithifilaria* sp. II and *C. grassi* [10, 37], in Greece with *C. grassii* [10, 26] and in Spain and Portugal with *C. grassii* and *Cercopithifilaria* sp. II [38]. Despite other *Cercopithifilaria* spp. not being detected in our study, they could be present in domestic dogs and/or wild canids of Iran. For example, DNA of *C. grassi* has been identified in one *Rhipicephalus* sp. III tick in the neighbouring country of Pakistan [14].

There was co-infection with other tick-borne pathogens in 17 of 32 dogs infected with *C. bainae*, based on PCR analyses of the blood of the infected dogs [42]. Specifically, 15 dogs were co-infected with *C. bainae* and *Hepatozoon canis*, and two dogs were infected with *C. bainae*, *H. canis* and *Anaplasma platys* [42]. Similar results have also been reported in southern Italy, where 58 *C. bainae*-infected dogs were simultaneously infected with at least one other tick-borne pathogen, such as *H. canis*, *A. platys* and *Babesia vogeli* [43]. The detection of *C. bainae* microfilariae indicates a prior tick exposure and should stimulate testing for other tick-borne pathogens. However, it has not yet been demonstrated whether *C. bainae* infection adversely affects the dog’s immune response to other pathogens [43].

Microfilariae of *O. lupi* were not detected in the examined samples, although 11.8% of the evaluated dogs showed mucosal discharges, conjunctivitis, keratitis or ulcers. Common ocular alterations of *O. lupi* in dogs are granulomatous mass on the scleral tissue, conjunctival swelling, keratitis, uveitis and mucopurulent discharge, as well as blindness [25]. Atypical migration of *O. lupi* in the larynx lumen of a dog has also been documented [44]. Cutaneous microfilariae of *O. lupi* have been reported in dogs and cats in Europe (e.g. Italy, Greece, Romania, Germany, Portugal, Spain, Switzerland), USA and Canada [45]. However, in areas outside of Europe and North America, such as Iran [24], Turkey [18, 22] and Tunisia [46], human cases of *O. lupi* are possibly associated with wild carnivores, which could play a role in the epidemiology of the infection [23].

In our study, the skin sediments of the examined cats and golden jackals tested negative for filarioids, probably due to the small sample size analysed. However, *O. lupi* has been previously reported in cats from Portugal and USA [47, 48]. On the other hand, the role of *C. aureus* in the epidemiology of *Cercopithifilaria* spp. and *Onchocerca* spp. is not known [49], advocating further research to elucidate the role of these animals as sylvatic carriers or reservoir hosts of these parasites.

All of the *C. bainae* isolated in the present study belonged to haplotype I. In contrast, a high genetic variability for *C. bainae* has been reported in previous studies conducted in Italy, Spain and Greece [10, 26, 50], suggesting a late introduction of the parasite from Europe. To date, 21 *cox*1 haplotypes of *C. bainae* have been identified [14, 26, 50], with haplotype I being the most commonly detected in dogs in the Mediterranean basin [50], as well as in *R. sanguineus* s.l. ticks from Italy, Greece, Malaysia, South Africa and USA, *R. turanicus* from Italy and *Rhipicephalus* sp. I from Greece and Italy [14, 26, 41]. The finding of three sequences displaying a relatively low nucleotide similarity (93.4%) with that of haplotype I (GenBank accession number: JF461457) is difficult to interpret, suggesting the existence of a different, as yet molecularly not characterised, species of *Cercopithifilaria*.

No information on the tick species circulating the microfilariae of *Cercopithifilaria* spp. in Iran is currently available. However, studies performed in Italy, Brazil, Spain, Portugal, Australia, Malaysia, Pakistan and South Africa reported *C. bainae* in different *Rhipicephalus* spp., indicating low specificity of these filarioids for ticks of this genus [14, 51, 52]. Therefore, further studies on dogs and tick vectors are needed to obtain a clearer picture of the geographical distribution of all three known *Cercopithifilaria* species infecting dogs in western Asia.

## Conclusions

This study reports for the first time *C. bainae* infection in dogs from Iran, expanding current knowledge on the geographical distribution of this parasite. Further studies on the different wild canine species (e.g., jackal, fox, wolf) and *Rhipicephalus* spp. ticks present in the country are needed to better understand the epidemiology of *Cercopithifilaria* spp. in the study area. Additionally, considering the previous human reports of *O. lupi* in Iran, more investigations on dogs as hosts should be performed with a focus on this zoonotic nematode.

## Supplementary Information

**Additional file 1: Video S1**.* Cercopithifilaria bainae* microfilaria during the microscopic examination of a skin sediment (×400 magnification).

## Data Availability

All data generated or analysed during this study are included in this published article and its additional files.

## Abbreviations

BLAST: Basic Local Alignment Search Tool
CI: Confidence interval
*cox*1: Cytochrome *c* oxidase subunit 1
ML: Maximum Likelihood
NaCl: Sodium chloride
NGO: Non-governmental organisation
s.l.: Sensu lato
